# Development and Prevention of Biofilm on Cochlear Implants: A Systematic Review

**DOI:** 10.3390/medicina60121959

**Published:** 2024-11-28

**Authors:** Alexios Tsikopoulos, Konstantinos Tsikopoulos, Konstantinos Sidiropoulos, Gabriele Meroni, Stefanos Triaridis, Lorenzo Drago, Paraskevi Papaioannidou

**Affiliations:** 1 1st Department of Pharmacology, School of Medicine, Faculty of Health Sciences, Aristotle University of Thessaloniki, 54124 Thessaloniki, Greece; kostastsikop@gmail.com (K.T.); ppap@auth.gr (P.P.); 2 School of Medicine, University of Patras, 26504 Patras, Greece; kcdroq@yahoo.gr; 3 Orthopedic Department, Eleni Dimitriou General Hospital of Florina, 53100 Florina, Greece; 4 One Health Unit, Department of Biomedical, Surgical and Dental Sciences, School of Medicine, University of Milan, 20133 Milan, Italy; gabriele.meroni@unimi.it; 5 1st Department of Otorhinolaryngology-Head and Neck Surgery, AHEPA General Hospital, Aristotle University of Thessaloniki, 54636 Thessaloniki, Greece; triaridis@hotmail.com; 6 UOC Laboratory of Clinical Medicine with Specialized Areas, IRCCS Multimedica Hospital, 20099 Milan, Italy; lorenzo.drago@unimi.it; 7 Clinical Microbiology and Microbiome Laboratory, Department of Biomedical Sciences for Health, University of Milan, 20122 Milan, Italy

**Keywords:** biofilm, cochlear implants, development, prevention, systematic review

## Abstract

*Background and Objectives:* Biofilm formation on cochlear implants (CIs) poses a major problem for surgeons, leading to a high incidence of explantation and revision surgery. Therefore, developing appropriate and cost-effective biofilm detection and prevention techniques is of the essence. In this systematic review, we sought to investigate the development of biofilm formation on CIs. We also elaborated on experimental preventative biofilm measures. *Materials and Methods:* We conducted a systematic search of both in vitro and in vivo literature published in PubMed, Scopus, and ScienceDirect, until 15 June 2024, for published studies evaluating the biofilm formation and strategies for inhibiting biofilm formation on CIs. Depending on the type of the included study, we assessed quality with the modified Consolidated Standards of Reporting Trials tool, the Joanna Briggs Institute Case Reports Critical Appraisal Tool, a modified Delphi technique, and the ROBINS-I tool. We synthesized the available information on biofilm formation on CIs and the infection prevention capacity of the included antibiofilm agents. *Results:* A total of 26 studies were included in this systematic review. Biofilms in CIs are usually localized in their recesses such as their removable magnet pocket as opposed to their smooth surfaces. *S. aureus* and *P. aeruginosa* are the most commonly isolated microorganisms, and they tend to be strong biofilm producers. The optimal treatment strategy for a biofilm-infected CI is explantation. Most of the examined biofilm prevention methods in CIs present sufficient antibiofilm activity. *Conclusions:* Biofilm formation in CIs is considered one of the most dreadful complications. There have been no specific guidelines for the treatment of those cases, with removal and/or replacement of the CI being the treatment of choice. Various experimental prevention methods provide promising antibiofilm activity both in vivo and in vitro.

## 1. Introduction

Cochlear implantation is a standard surgical treatment method for both pediatric and adult patients suffering from permanent sensorineural hearing loss of severe to profound level. In general, the infection-associated surgical complications are rare, ranging from 0.72% to 1.08% in children and adults, respectively [1]. Nevertheless, when post-operative infection occurs, it commonly leads to poor outcomes [2,3]. Although the evolution of surgical techniques has significantly reduced complications [4,5], persistent infections or progressive flap breakdown usually require explantation of the implants and subsequent replacement [6]. To elaborate further, due to the presence of biofilms, administration of antibiotics is more often than not insufficient to eradicate the infection [7] and as a result explantation and revision surgery are required [8]. At present, there are no widely accepted guidelines for the management of CI infection. However, the most reliable solution to this problem includes the removal of the device which inevitably leads to increased healthcare costs and morbidity.

Like any other implantable devices, CIs are susceptible to biofilm development as they provide a surface for microbial colonization and growth [9,10,11,12]. The formation of a biofilm starts with the adherence of individual planktonic bacteria to a solid surface [13]. Following this, the bacteria communicate and coordinate their actions through a mechanism referred to as quorum-sensing [14], leading to the formation of a colony when a critical mass of bacteria is reached. Biofilms act as reservoirs, being able to release individual planktonic bacteria into the surrounding tissue and therefore leading to resilient infections despite intensive antimicrobial therapy [15].

In this systematic review, we aimed to describe the development and prevention modalities of biofilm formation on patients with CIs, presenting the causative microorganisms and the major infection-related outcomes of each study.

## 2. Methods

We conducted this systematic review according to the Preferred Reporting Items for Systematic Reviews and Meta-Analyses (PRISMA) guidelines [16].

### 2.1. Eligibility Criteria

We included exclusively studies assessing the formation and prevention of biofilms on CIs, both in vivo and in vitro. The included in vivo studies were conducted either on human beings or on animals. No further exclusion criteria were applied. Every type of study except for reviews was qualified for qualitative synthesis.

### 2.2. Literature Search

Two reviewers (A.T. and K.T.) independently conducted the literature search for published studies examining the formation and prevention of biofilms on CIs. The databases of PubMed, Scopus, and ScienceDirect were comprehensively assessed until 15 June 2024. Furthermore, reference lists from relevant articles were manually searched to identify additional studies. It is of note that only studies in English were assessed. The search strategy included the terms: “biofilms” and “cochlear implants” and (“prevention” or “formation” or “development”). In the case of discrepancies between authors in selecting articles, they were resolved via discussion.

### 2.3. Selection of Studies

Potentially relevant records were identified through the literature search by two reviewers (A.T. and K.T.) independently. Following deduplication, the titles and abstracts of the remaining studies were screened for eligibility. The full text of the remainder of the articles was evaluated for possible inclusion against our eligibility criteria.

### 2.4. Data Extraction

Two researchers (A.T. and K.T.) independently extracted data regarding the author, the year, the country of publication, the microbial species, and the infection-related outcomes. For the articles assessing biofilm formation, we also presented the main findings of the studies, and for those evaluating the prevention of biofilm formation, we presented the available prevention methods.

### 2.5. Quality Assessment

The two investigators (A.T. and K.T.) independently evaluated the quality of the enrolled in vitro and animal studies by utilizing the modified Consolidated Standards of Reporting Trials’ risk of bias instrument [17]. The quality of the included case reports was evaluated using the Joanna Briggs Institute Case Reports Critical Appraisal Tool [18], whereas for the quality assessment of the case series, the Moga score [19] was implemented. More specifically, as per the Modified Delphi Panel, we used an 18-criterion checklist. Finally, regarding the quality appraisal of prospective cohort, we utilized the ROBINS-I tool [20].

## 3. Results

### 3.1. Study Selection

**Figure 1:**
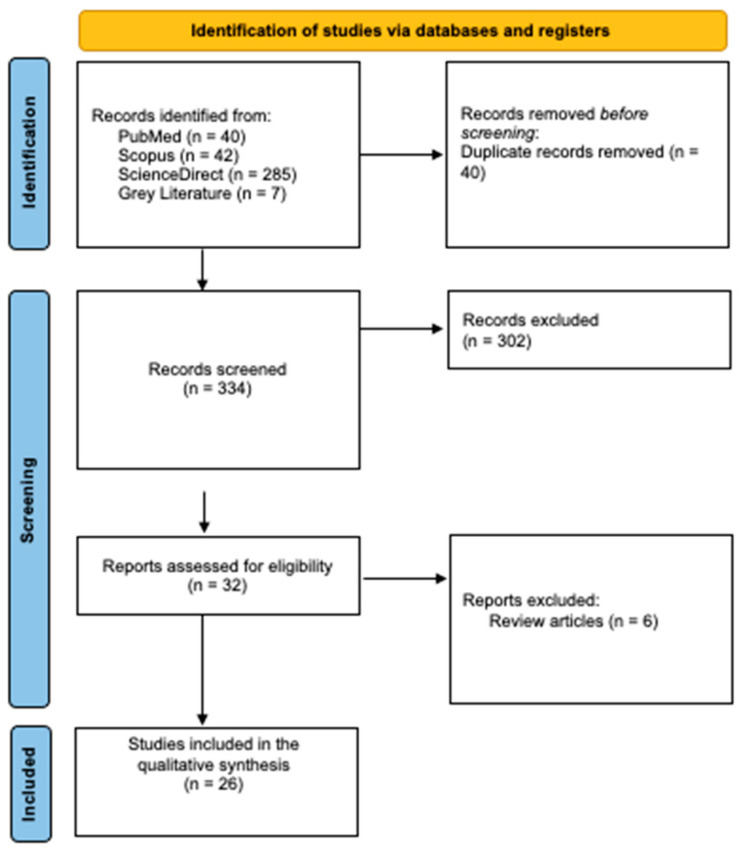
PRISMA 2020 flow diagram.

The initial literature search yielded 374 potentially relevant studies. Following deduplication, the eligibility of the remaining studies was assessed according to their title and abstract data resulting in 32 articles that were deemed relevant and subjected to full-text screening. Finally, 26 studies were considered to be eligible for qualitative synthesis. The overall research strategy yielded 15 results for studies regarding development of biofilms on CIs and 11 studies regarding possible prevention methods of biofilm formation on CIs (Figure 1).

### 3.2. Study Characteristics

The included studies were published between 2004 and 2021 (Table 1 and Table 2). In total, seven studies were conducted in USA [10,11,21,22,23,24,25], two in India [26,27], one in Denmark [28], one in Turkey [29], one in South Korea [30], two in France [31,32], one in Switzerland [33], two in Israel [34,35], one in Romania [36], one in Austria [37], one in the Czech Republic [38], two in Germany [39,40], two in the UK [7,41], and two in Australia [42,43]. Nine of the included studies regarding biofilm development were case reports [11,21,23,25,27,30,31,33,38,41], three of them case series [2,24,28], one of them a prospective cohort study [32], and one an in vitro study [10]. Regarding biofilm prevention, seven were in vitro studies [15,34,35,37,39,40,42], three were animal studies [22,29,36], and one was a case series [26].

### 3.3. Quality Assessment

Only a few of the in vitro studies reported the calculation method of sample size, randomization determination, allocation concealment mechanisms, implementation details, registration protocols, and blinding. Contrariwise, most studies provided the background and objectives, methods determination of interventions, outcome presentation and estimations, statistical methods, and results presentation (Supplementary File S1). Furthermore, the case reports were assessed with yes responses for every required category (Supplementary File S2). For case series assessment, no study was awarded with 14 or more yes responses (≥70%). Thus, they could not be characterized as high-quality (Supplementary File S3). Finally, the unique prospective cohort study was deemed of low risk of bias (Supplementary File S4).

### 3.4. Development of Biofilms on Cochlear Implants

It was Antonelli et al. [24] who first sought to prove the presence of biofilms on the surfaces of extruding CIs, to explain the limited efficacy of the administration of antibiotics for the management of CI infections. Using scanning electron microscopy (SEM), they achieved the direct visualization of bacterial biofilms on the surface of infected CIs. Generally, they suggested that after suspicion of implant–skin flap infection and biofilm formation, there is a definite indication of performing SEM. In total, two CIs were removed due to device infections from patients, two CIs were removed because of failure, and two CIs were never implanted and served as controls. On the surface of CIs either infected or removed due to device failure, bacteria and amorphous extracellular debris were detected. However, biofilm formation was considered definite in only one of the infected and possible in the rest explanted devices. The never-implanted controls were microbially contaminated but were not characterized by the formation of exopolymeric matrix, suggesting contamination without biofilm formation. The isolation of *S. aureus* in both infected CIs, which is an uncommon middle ear pathogen, indicated a non-otologic source, leading to the assumption that most staphylococcal infections derive from skin flora.

One year later, Pawlowski et al. [11] attempted to assess the traits of bacterial biofilm on CIs. For this study, they initially removed surgically the receiver stimulator package of a CI from a patient to prove the development of biofilm on its surface. The extracted receiver stimulator was subjected to culture and SEM for the confirmation of the presence and evaluation of distribution of biofilm, with the major pathogen in it being *S. aureus*. Other bacterial species apart from Staphylococcus were not isolated. SEM examination revealed a layer of yellow-colored extracellular polymeric substances, indicative of *S. aureus*-containing biofilm. Of note is that the depressions on the surface of the implant presented the greatest biofilm density.

Vaid et al. [27] reported on another case of biofilm formation on an infected and finally extracted CI at a 2-year-old patient. The patient presented five months after the implantation with recurrent swellings in the post-auricular area. The per os administration of antibiotics could not prevent the ulceration and rupture of the affected area and following the exposure of the receiver stimulator package. The latter was covered with a slimy substance, whereas the electrode array in the mastoid was unaffected. The microbiological evaluation of the CI provided strong evidence of biofilm formation in the removable magnet pocket. Similarly with the previous studies, *S. aureus* was identified as the main pathogen.

The most extensive prospective study about biofilm formation on CIs was conducted by Olsen et al. [28]. They examined the biofilm formation on 653 implantations executed on 506 adult patients. Of all the implanted devices, 63 of them were infected postoperatively, with 52 of them being characterized as minor and 11 of them as major infections. Overall, the rate of major infections was 2% and 73% of them were finally explanted. Biofilm was detected in 8/11 (73%) of the major infections, which led to the explantation of 7/8 (88%) of them. In the cases of major infections, the main pathogen cultivated in 5/8 (63%) of them was *S. aureus*, whereas other pathogens such as *P. aeruginosa*, non-pathogenic diplococci, or even no growth was detected in the remaining three, accounting for 13% each. In 17/52 (33%) of the minor infections, in 2 cases, *S. aureus* was cultivated, and no growth was detected in the other 15 cases. Generally, this study associates the existence of biofilm on the surface of a CI with a severe risk of its explantation ultimately.

Im et al. [30] reported biofilm formation on the magnet of a CI at a pediatric patient, which suffered from a methicillin-resistant Staphylococcus aureus (MRSA) infection. The SEM analysis of the MRSA biofilms from the surface of an explanted CI revealed biofilms with three-dimensional extracellular polymeric matrix. At the margins, the biofilms were solitary and scattered, whereas centrally they were large and plate-like, forming towers and water channels.

Moreover, Celerier et al. [31] executed a retrospective review of children subjected to cochlear implantation with complaints about atypical pain in the retro-auricular area postoperatively. Overall, 20 out of 1.448 (1.4%) implanted patients were included. A microbiological culture of soft tissues obtained during CI explantation for 10/20 cases, 2 magnets and 8 CI, gave positive results for 2 explanted CIs and for 1 magnet. SEM proved biofilm formation in six explanted CIs and on one explanted magnet. In total, 5/7 biofilms, 6 from CIs and 1 from the magnet, were positive for *S. aureus* and *P. aeruginosa*. The localization of the biofilms was mainly on the magnet, on the silicone magnet pocket, in the emergence of the electrode array from the receiver stimulator package, or on the extra-cochlear electrode plate. Hence, pain after cochlear implantation could be directly associated with the existence of biofilms on CI.

Biofilms can also be detected in dysfunctional CIs without signs of infection. To be more specific, the study of Ruellan et al. [32] was the first to prove the existence of biofilms on the surface of electrode array of CIs after explantation due to failure of devices without signs of infection, and thus could be considered a landmark for biofilm research in CIs. The examination using SEM and confocal laser scanning microscopy (CLSM) ascertained the existence of bacterial biofilm. The existence of bacterial biofilm in the middle-ear part of the electrode array was confirmed in one specimen, whereas in the inner-ear part of electrode arrays they did not detect biofilms.

Additionally, Cristobal et al. [23] were the first to present fungal infection of a CI at a child with chronic suppurative otitis media and bilateral ventilation tube placement despite being managed with perioperative antibiotics. Despite the administration of oral cefdinir and topical ofloxacin, a Candida biofilm was detectable on the surface six weeks after implantation. The results of SEM proved the existence of a fungal biofilm on the CI receiver and the electrode array.

Furthermore, Loeffler et al. [10] created an in vitro model to examine the process of biofilm development. They constructed silastic models which represented CIs with or without a magnet pocket and additionally, with or without a titanium blank in the pocket. CIs were cultured with a biofilm forming strain of *S. aureus*. For the assessment of the adherence of planktonic bacteria and of the development of biofilm, they applied SEM and quantitative bacterial counts as well. The results of the study showed that in the CI models without a titanium blank in the magnet pocket, the number of adherent bacterial was significantly higher than those with a titanium blank (*p* = 0.0097). What is more, a significantly lower biofilm formation was recorded in the CI models without a magnet pocket (*p* = 0.0121).

Kos et al. [33] applied immune microscopy for the assessment of biofilm formation in another patient with a *S. aureus* infection on a CI. They noticed that polysaccharide intercellular adhesion was achieved on the surface of the CI. However, initial bacterial colonization was more profound in the pocket of the removable magnet, and colonies of *S. aureus* adhered to the electrode wire without biofilms. The persistent *S. aureus* infection of the CI failed to be treated with conservative treatment and was finally extracted.

Moreover, Skrivan et al. [38] presented a case report of a pediatric patient with CI and surgical site infection because of acute otitis media. Following an episode of purulent otitis media, recurrent abscess formation over the receiver–stimulator body occurred. A gelatinous mass on the receiver–stimulator was noticed, and a biofilm presence was highly suspected. Bacteriological examination revealed the presence of Haemophilus influenzae. After conservative management including incision of inflamed skin and wound debridement, it was decided to explant a functioning device. This case strengthens the notion that especially in the event of biofilm formation, it is necessary to remove the infected functioning device.

Another interesting study derived from Asfour et al. [25]. They investigated the application of fluorescence in in situ hybridization (FISH) and microbial community profiling (MCP) for the study of microbial environments of explanted devices. In total, 12 explanted devices underwent microbiological analysis, 5 of which were explanted because of suspected implant associated infection. FISH analysis revealed biofilm presence on all infected devices and only partial correlation of cultures with biofilm composition. MCP analysis correlated with cultures of infected devices and suggested a diverse microbial composition of explanted devices.

Biofilm formation on CIs has also been associated with fulminant meningitis. In more detail, Makarem et al. [21] investigated the case of an implanted 2-year-old infant with dysplastic inner ear who died of bacterial meningitis. The explanted cochlear implant was studied initially with a SEM and later was subjected to in situ hybridization in search of bacterial DNA. SEM revealed cellular formations on the surface of the CI, which later bind to the probe used for the in situ hybridization, evidence suggestive of biofilm formation on the CI surface.

Fishpool et al. [41] were the first to report an ultrasonication technique on an explanted CI to demonstrate biofilm formation. Instead of using SEM, they placed the explanted CI in a nutrient broth in an ultrasonic water bath. The implant was ultrasonicated for 5 min and the broth was cultured on blood agar, incubated aerobically and anaerobically for 48 h, and then incubated in air on Maconkey agar for 24 h. In this way, it is generally possible to improve the recovery of organisms from the explanted device, allowing their identification. In this case, a heavy confluent growth of *S. aureus* was obtained from the sample collected after ultrasonication. The mechanism for this is the release of organisms trapped in the gluelike glycocalyx biofilm matrix surrounding the device.

Last but not least, Chen et al. [43] applied SEM to assess biofilm characteristics following cochlear implantation. For this study, 1251 implanted patients were enrolled. Skin flap infection (SFI) after cochlear implantation was reported in 16 patients (1.28%), and most of them happened in patients younger than 6 years old. In total, 50% of flap infections were attributed to *S.aureus*. Using SEM, they successfully confirmed the formation of bacterial biofilm on the surface of CI in every SFI patient.

Overall, the causative microorganisms of biofilm formation on CI in most of the cases was *S. aureus*. However, other pathogens such as *H. influenza* and *P. aeruginosa* or even candida species should be considered less common culprits on infected devices as well.

### 3.5. Prevention of Biofilm Formation on Cochlear Implants

#### 3.5.1. Coating with ZnO and MgF_2_ Nanoparticles

Natan et al. [35] applied nanoparticles (NPs) for the inhibition of biofilm formation on CIs. To achieve this, they sonochemically coated CIs with ZnO or MgF_2_ NPs on the same platform to assess their synergy against *S. aureus* and *S. pneumoniae*. SEM analysis demonstrated that ZnO and MgF_2_ were uniformly distributed on the surface of CI with average sizes of 200–300 nm and 25 nm, respectively. The application of ZnO-MgF_2_ on the surface of CIs significantly decreased the biofilm density of the two examined pathogens.

#### 3.5.2. Zwitterionic Polymer/Polydopamine Co-Deposited Coating

Chen et al. [42], in an attempt to solve the problem of bacterial biofilm formation on CIs developed a zwitterionic polymer/polydopamine co-deposited coating with anti-bacterial and anti-biofilm properties via CuSO_4_/H_2_O_2_ trigger. This trigger system not only accelerated the co-deposition rate but also enhanced the anti-bacterial properties. In more detail, the copper-containing zwitterionic coating consisted of anti-adherent poly-sulfobetaine-methacrylate (PSB) and steadfast-polydopamine (PDA). The addition of CuSO_4_/H_2_O_2_ accelerated this co-deposition reaction. In the in vitro and in vivo studies, the PSB/PDA(Cu) coating exhibited excellent anti-biofilm properties against representative gram-positive and gram-negative bacteria.

#### 3.5.3. Polydimethylsiloxane (PDMS) Casing of CIs

Furthermore, Goldfinger et al. [34] evaluated the efficacy of two different coatings for the polydimethylsiloxane (PDMS) casing of CIs. The coatings were made by either liquid phase deposition (LDP) or atomic layer deposition (ALD) and were based on thin titania films. Two different detection assays were applied for the assessment of the antibacterial potential of PDMS coating with titania: BCA protein and CLSM. They examined not only the inhibition of attachment by planktonic bacteria, but also the subsequent biofilm formation, with the examined bacteria being *E. coli*. First, after applying the BCA protein assay, they comparatively assessed the biofilm development on PDMS samples with and without a titania coating. The titania-coated surfaces presented a reduction in biofilm formation with protein quantity on ALD- and LPD- treated samples by 44 and 41%, respectively. By applying CLSM, the authors confirmed further these results, showing a biofilm reduction of 91 and 89% for ALD- and LPD-coated surfaces, respectively. There were statistically significant differences between the uncoated PDMS surface and both ALD- and LPD-titania-coated PDMS coupons.

#### 3.5.4. Antibacterial Polysiloxane Polymers and Coatings for CIs

Cozma et al. [36] assessed the efficacy of antibacterial polysiloxane polymers and coatings for the prevention of biofilm formation on CIs. To do so, the authors sought to create membranes resistant to bacterial adherence and biofilm formation. They synthesized new materials based on polysiloxane modified with different ratios of N-acetyl-L-cysteine (NAC) and crosslinked via UV-assisted thiolene addition. The membranes were initially tested in vitro for the evaluation of microbial adherence against *S. pneumoniae*. For the in vivo testing, they implanted WISTAR rats for 4 weeks with crosslinked siloxane samples without and with NAC. After the in vivo tests, very small numbers of bacteria were noted on the material surface without signs of biofilm formation.

#### 3.5.5. Biofilm Formation in CIs with Cochlear Drug Delivery Channels

Johnson et al. [37] assessed the formation of biofilms on drug delivery (DD) ports when subjected to different types of penetration. For starters, in order to represent CIs with a DD channel, the authors created silastic models with either an intact or a widely opened port, and additionally with either a non-coring needle penetrating the port, or a non-coring needle removed from the port. CIs were exposed to a culture of a biofilm-forming strain of *S. aureus* for 5 days, and following this, they assessed the biofilm formation with quantitative bacterial counts and SEM. The study showed that CIs with widely fenestrated ports presented significantly higher bacterial counts (*p* = 0.0003).

#### 3.5.6. The Effect of a Novel Quorum-Sensing Inhibitor Against Biofilm Formation on CIs

In addition, Cevizci et al. [29] sought to evaluate the antibiofilm properties of a novel quorum-sensing inhibitor, named “yd 47”, on CIs. For this purpose, CIs were cut into small pieces and were implanted subcutaneously in the retro-auricular area of two guinea pigs, bilaterally. The examined pieces in the study and control sides were implanted in *S. pneumoniae* strain solution and saline, respectively. The animals were administered “yd 47” intraperitoneally twice daily for a period of three months. The guinea pigs were clinically examined with palpation and inspection, without any sign of implant infection. No biofilm formation by pneumococci was detected in either the study and control implant materials and at the surrounding soft tissue.

#### 3.5.7. Use of Betaine Surfactant and Polyhexanide Against Biofilm Formation

Suri et al. [26] were the first to report a biofilm prevention method on CIs using betaine surfactant and polyhexanide. They made a protocol for the management of biofilm after salvaging CIs. They noticed that no debridement nor cleansing procedure was able to fully remove biofilm since they still were able to regrow within days. The application of polyhexanide and betaine surfactant reduced surface tension and significantly removed debris and bacteria. They concluded that the coverage of the bed and flap with gel prevented the further growth of biofilm, and it was considered an efficient modality for the prevention of device-associated *P. aeruginosa*, *S. aureus,* and *S. epidermidis* infection.

#### 3.5.8. Treatment with Terpinen-4-Ol or Hydrogen Peroxide

Brady et al. [15] isolated and cultured a methicillin-sensitive *S. aureus* from a biofilm growing on an extracted CI. At a first phase, they determined the susceptibility of this bacterial isolate, either when grown planktonically or in a biofilm, to a range of conventional antibacterial agents and to tea tree oil and its active component terpinen-4-ol. 1% tea tree oil did not completely prevent the formation of biofilm at 1 h whereas 5% tea tree oil managed to completely eradicate the biofilm development by the CI methicillin-sensitive *S. aureus* isolate, demonstrating that sufficient eradication of bacterial biofilm could be achieved by the administrating terpinen-4-ol or hydrogen peroxide for as little as 1 h.

#### 3.5.9. Surface Modification by a Layer-by-Layer Polyelectrolyte Assembly Method

Moreover, in order to address the issue of biofilm formation on CIs, Kao et al. [22] speculated that surface charge modification could result in the inhibition of *P. aeruginosa* biofilm development on CIs not only in vitro but also in vivo. To begin with, for the investigation of the efficacy of surface charge in vitro, they modified the surface wells in culture plates using a layer-by-layer polyelectrolyte assembly method. They evaluated the level of bacterial adherence after 30, 60, and 120 min. In addition, for the in vivo examination of the effect of surface charge modification, a similar modification of the surface of titanium microscrews took place and following the microscrews were surgically implanted into the dorsal calvaria of adult rats and inoculated with bacteria. Upon examination two weeks after the surgical implantation, the remaining bacteria presented a significant in vitro decrease in adherence, which was achieved by surface charge modification of the microscrews.

#### 3.5.10. S53P4 Bioactive Glass

Höing et al. [39] investigated the hypothesis that the coating of S53P4 bioactive glass on CIs surfaces could lead to a reduction in biofilm formation on them, and differs between bacterial species. To elaborate, the biofilm formations of *Pseudomonas aeruginosa* (ATCC9027), *Staphylococcus aureus* (ATCC6538), *Staphylococcus epidermidis* (ATCC12228), and *Streptococcus pyogenes* (ATCC19615) on 17 different surfaces were tested. Every tested microbial species formed biofilms on the CI surfaces under examination in a strain-dependent manner. In more detail, *S. aureus* demonstrated a significantly higher biofilm formation on metal components in comparison with silicone, whereas the rest of the strains did not present a material specific biofilm formation. Following this, they reported that the coating of S53P4 bioactive glass led to a significant reduction of *P. aeruginosa* and *S. aureus* biofilm since the four tested bacteria species exhibited biofilm formation on the covered CI surfaces in a species- and material specific manner. Another study examining the effects of bioactive glass on CIs executed by Kirchhoff et al. [40], also confirmed that the capability of *P. aeruginosa* and *S. aureus* for the formation of biofilm on CI component materials was different with and without the coating of bioactive glass. S53P4 bioactive glass caused significant changes in the morphology of biofilm that could be visualized by SEM for the same bacteria species.

## 4. Discussion

CIs are not usually complicated by a microbial infection. However, in case of infection, it is considered one of the most dreadful complications. These infections pose a challenge for clinical doctors since they are resistant to treatment with conventional antibiotics. The data regarding proper management of these cases are not concrete, and patients are usually subjected to explantation and revision surgery [2]. This situation is attributed to biofilm formation on CIs [2,10,11,23,24,27,28,30,31,32,43]. CIs can potentially provide surfaces for bacterial biofilm formation like any other biomaterial [11]. This high resistance to antibiotic therapy of biofilms is attributed to their structure, since not only does the extracellular polysaccharide matrix reduce antibiotic penetration but the deeper regions of a biofilm are also anaerobic with slow-growing cells.

A dilemma faced by almost every CI surgeon, in the absence of concrete management guidelines, is whether to consider antimicrobial therapy or to immediately explant the device when confronting biofilm infections in CIs. Olsen et al. [28] in a retrospective study, reported an infection rate of CIs of 2% and an explantation rate after major infections of the devices of 73%. Biofilm formation was detected in 88% of the explanted CIs. These percentages were equal to the reported rated by Cunningham et al. [2] for the adult population. In their study, the major infection rate was 3%, and the explantation rate was 1.3%. Other authors as well have correlated the biofilm development in implant-related infections with explantation ultimately. Vaid et al. [27] from their experience suggested that antimicrobial treatment has a limited role in patients with flap breakdown, and they recommend early CI explantation in these patients. However, in early-diagnosed patients with no overlying skin breakdown, the administration of wide spectrum antibiotics could be effective for handling biofilm-related infections [27]. Although no definitive conclusions can be reached from the studies above, it could be speculated that the formation of biofilm on CIs is directly correlated with a high risk of explantation [28]. However, the relatively low rates of explantation support that cochlear implantation should be considered a safe procedure which entails minimal risk of extracting the device because of postoperative infection.

Other unspecific symptoms, such as pain, could be attributed to biofilm formation on CIs as well. Celerier et al. [31] reported pain as a complication of biofilm formation on a CI, a condition that should be not be considered unusual since it is recorded in more than 1% of the implanted cases. Similarly, Todd et al. [44] reported that in 5/581 patients implanted between 1992 and 2013, pain was the chief complaint, while the auditory performances of the implanted patients were not affected. In addition, for Chung et al. [45] pain was a common sign related with soft tissue failures. In contrast with this evidence, in a literature review executed by Terry et al. [46], pain was reported as a plausible complication in none of the 88 articles published between 2003 and 2013.

Surprisingly, biofilms can also be detected in dysfunctional CIs without signs of infection. Ruellan et al. [32] investigated the biofilm formation on CIs from nine adults patients explanted due to failure without evidence of infection. They were the first to report biofilm formation on the surface of the electrode array of explanted CIs because of device failure in the absence of an infection. Utilizing both SEM and CLSM, they found one case out of the nine explanted CIs with a bacterial biofilm on the electrode array.

From a microbiological standpoint, the existing data indicate that *S. aureus* and *P. aeruginosa* are the main pathogens for biofilm-related implant infections [23,24], followed by *S. pyogenes* and *S. epidermidis* [39] as well as *E.Coli* [2]. In general, there is an obvious prevalence of staphylococcus [10,11,31,43]. Like in other studies, Olsen et al. [28]. reported that the most common pathogen was *S. aureus*. It was cultivated in 63% of the major infections with biofilm and the remaining cases displayed *P. aeruginosa*, non-pathogenic diplococci, and no growth, accounting for 13% each. Moreover, Im et al. [30] reported biofilm formation on a CI magnet from a MRSA infection. Additionally, Cristobal et al. [23] presented the first report of fungal biofilm formation on a CI. Biofilms could derive from several sources. For instance, Cain et al. [47] suggested the retrograde flow of bacteria through the eustachian tube as a possible route of bacterial CI contamination postoperatively. In another infectious case described by for Kanaan et al. [48], there was a prevalence of *P. aeruginosa* and bacteria from the teeth in biofilm bacterial spectrum. Nevertheless, the isolation of *S. aureus*, an uncommon middle ear pathogen, in most of the CI infections, indicates a non-otologic source. Hence, most staphylococcal infections presumably develop from skin flora. It is of note that, cochlear implantation should be considered safe in middle ears with chronic contamination [49] and intermittent acutely infection [50] since the rate of postoperative device infection is low. Finally, the lack of reported cases of fungal infections of CIs, indicates that colonization during cochlear implantation is not likely. In the unique case described, the fungal infection may have derived from the colonized external auditory canal, ventilation tubes, or possibly adenoid pad [51].

Highly important is the observation that biofilm formation on CIs is more prevalent in the depressions along their surface. To elaborate, recesses such as the removable magnet pocket seem to harbor more biofilm [10]. Indeed, SEM analysis of infected CIs after explantation have proven that the highest biofilm growth occurs in surface defects and in the opening of the magnet pocket [11]. This observation could be explained by the presence of a pocket for a removable magnet, which creates a “dead-space” that promotes biofilm development by protecting microorganisms from any fluid flow and from the host immune response.

Loeffler et al. [10] confirmed the hypothesis that the magnet pockets of CIs potentially increase the risk of biofilm formation. They noted a significantly higher biofilm development in the CI models with simulated magnet pockets regardless of the existence of a “magnet” in the pocket. However, biofilms may also extend between the magnet and the silicone to the medial side of the magnet [11]. Similarly, accumulation in the depressions for the electrode contacts was reported in a study regarding fungal biofilm on a CI [23] even though SEMs of electrode arrays from infected CIs did not show significant biofilm formation at the electrode–silastic interface [11,24]. Additionally, extra caution should be addressed regarding magnet strength as these might reduce vascularization of the skin at the receiver location and especially in fragile skin, such as in elder patients. This could result in flap breakdown, infection, bacterial biofilm formation, and explantation. Extra attention should be given when patients complain about painful magnet locations on the skin. In this case, magnet strength should be lowered and the patient should temporarily not wear the device for skin recovery.

Prevention might be the key to the confrontation of biofilm infections in CIs. Two should be the major concepts of the preventative strategies: the modification of the shape of the CIs to avoid as many risky areas as possible and the development of novel antibacterial materials or coatings for these devices. Other than that, it is self-understood that intra- and peri-operative treatment plays a major role in the prevention of biofilm infections in CIs. Additionally, as has been stated above, magnet strength is a factor that affects the biofilm formation on CIs as well. Overall, a common denominator of the proposed biofilm prevention methods is the fact that in most cases, their efficacy is expected to be tested clinically in humans.

### 4.1. Strengths and Limitations

The current systematic review not only presents the current literature on biofilm formation on CIs thus far but also offers an overview of the novel biofilm inhibition methods that have been tested over the last decades, with the data stemming from a total of 22 studies. Although the biofilm prevention methods tested in the present study demonstrated satisfactory results, it is highlighted that a minimum biofilm inhibition threshold of 80% should be reached compared to control groups for the results to be clinically meaningful [45]. In most of the included studies, the biofilm reduction was not quantified and therefore safe conclusions cannot be reached based on those data. Another limitation in the current study is the fact that the evidence regarding biofilm prevention stems from in vitro or animal studies, thus introducing some uncertainty in the results. Last but not least, we wish to highlight that in the current systematic review, we utilized evidence stemming from various levels of evidence, which could have resulted in heterogeneity in the results.

### 4.2. Implication for Future Research

We wish to underline that the safety of the methods tested in the source studies of the current systematic review has not been thoroughly investigated in vivo as of yet. However, we advocate that further research assessing the safety profile in vivo be conducted in the foreseeable future to allow for those implant modifications to be gradually implemented into clinical practice. Provided that those methods are safe and effective for use in living organisms, the proposed coatings could be then made available on the market. To achieve this objective, large-scale trials should be conducted to corroborate the findings of the present systematic review.

## 5. Conclusions

Biofilm formation on CIs leading to recalcitrant infections is a major complication, associated with high morbidity. The biofilm communities are well-known to be strongly resilient to host immune responses and antibiotic therapy, rendering the management of those cases a challenge for the clinical doctors [52] since the microorganisms embedded in a biofilm are protected by a polymer matrix and have lower growth and oxygen consumption rates [53]. As of yet, there have been no specific guidelines for the treatment of those cases, with removal and/or replacement being the treatment of choice. The most important conclusion regarding the available literature and incidence of the mentioned complication (around 1%) is that if a situation of an infected implant with bacterial microfilm occurs, explantation is the only reasonable and best-suited treatment strategy. The interest of the scientific community should focus on the prevention of this complication.

It is of note that the discovery that biofilms are directly associated with CIs infection influences the design parameters of the devices. To be more specific, biofilms predominantly develop in surface depressions and pockets along the surface of CIs [24]. From a microbiology point of view, *P. aeruginosa* and *S. aureus* biofilms are the most commonly isolated pathogens found on explanted CIs [11]. Various prevention methods against the biofilm formation on CIs have been suggested in recent decades, and their efficacy in most cases is expected to be tested clinically.

## Figures and Tables

**Table 1:** Biofilm formation on cochlear implants.

Study (Year)	Isolated Microbial Species	Infection-Related Outcomes	Main Findings
Antonelli (2004) [24]	*S. aureus*	Indication for direct visualization of bacterial biofilms on the surface of infected CIs using SEM after suspicion of implant-skin flap infection.	Biofilm formation on the surface of 1/4 CIs, either infected or removed due to device failure.
Pawlowski (2005) [11]	*S. aureus*	Biofilm formation on a surgically removed receiver stimulator package of a CI.	The depressions on the surface of the receiver stimulator package present the greatest biofilm density.
Vaid (2013) [27]	*S. aureus*	Recurrent swellings of the post-auricular area are indicative of biofilm formation on the receiver stimulator package.	Biofilm formation on a surgically removed receiver stimulator package of a CI. Unaffected electrode array.
Olsen (2018) [28]	*S. aureus*, *P. aeruginosa*, non-pathogenic diplococci	Association of the existence of biofilm on the surface of a CI with a severe risk of its ultimate explantation.	Biofilm was detected in 8/11 (73%) of the major infections. Explantation of 7/8 (88%) of them.
Im (2015) [30]	MRSA	Biofilm formation on the magnet of an explanted CI from a pediatric patient.	Biofilm formation on the magnet of a CI caused by MRSA infection.
Celerier (2017) [31]	*S. aureus, P. aeruginosa*	Direct association of atypical pain in the retro-auricular area postoperatively after cochlear implantation with the existence of biofilms on CI.	Out of 20 implanted patients with retro-auricular pain, biofilm formation in 6 explanted CIs and on 1 explanted magnet. Localization of the biofilms mainly on the magnet, on the silicone magnet pocket, in the emergence of the electrode array from the receiver stimulator package, or on the extra-cochlear electrode plate.
Ruellan (2010) [32]	N/A	Detection of biofilms on the surface of electrode array of CIs in dysfunctional devices without signs of infection.	Bacterial biofilm formation in the middle-ear part of the electrode array in one CI after explantation due to failure of device without signs of infection.
Cristobal (2004) [23]	Candida species	Confirmation of fungal biofilm formation of a CI.	Existence of a fungal biofilm on the CI receiver and the electrode array upon explantation six weeks after implantation.
Loeffler (2007) [10]	*S. aureus*	Association of magnet pockets with higher biofilm formation in the CI models.	Significantly higher number of adherent bacterial in CI models without a titanium blank in the magnet pocket (*p* = 0.0097). Significantly lower biofilm formation in the CI models without a magnet pocket (*p* = 0.0121).
Kos (2009) [33]	*S. aureus*	Confirmation of presence of bacterial biofilm in the pocket of the removable magnet.	More profound biofilm formation in the pocket of the removable magnet. Adhesion of bacteria to the electrode wire, without formation of biofilms.
Chen (2022) [43]	*S. aureus*	Direct association of skin flap infection after cochlear implantation with biofilm formation.	Bacterial biofilm formation on the surface of CI in 16 patients with skin flap infection.
Skrivan (2016) [38]	*H. influenzae*	Biofilm formation on the receiver-stimulator body following purulent otitis media.	Explantation of functioning device because of biofilm infection.
Asfour (2022) [25]	N/A	Application of FISH and MCP for the study of microbial environments of explanted devices.	FISH analysis revealed biofilm presence on all infected devices and only partial correlation of cultures with biofilm composition. MCP suggested a diverse microbial composition of explanted devices.
Fishpool (2012) [41]	*S. aureus*	Utilization of an ultrasonication technique on an explanted CI to demonstrate biofilm formation.	Identification of the released through ultrasonication organisms trapped in the gluelike glycocalyx biofilm matrix surrounding the CI.
Makarem (2008) [21]	N/A	Bacterial meningitis due to biofilm formation on CI in a child dysplastic ear.	SEM revealed cellular formations on the surface of the CI which later bind to the probe used for the in situ hybridization, evidence suggestive of biofilm formation on the CI surface.

Abbreviations: CI = cochlear implant, MRSA = methicillin-resistant Staphylococcus aureus, FISH = fluorescence in situ hybridization, MCP = microbial community profiling.

**Table 2:** Prevention of biofilm formation on cochlear implants.

Title (Year)	Prevention Method	Microbial Species	Infection-Related Outcomes
Natan (2016) [35]	Coating with ZnO or MgF_2_ NPs	*S. aureus, S. pneumoniae*	Significant decrease in the biofilm density
Chen (2023) [42]	Zwitterionic polymer/polydopamine co-deposited coating via CuSO_4_/H_2_O_2_ trigger	Gram-positive and Gram-negative bacteria	Excellent anti-biofilm properties
Goldfinger (2014) [34]	Coatings by either LDP or ALD, based on thin titania films	*E. coli*	Biofilm reduction of 91 and 89% for ALD- and LPD-titania- coated surfaces PDMS coupons, respectively., compared with uncoated PDMS
Cozma (2021) [36]	Coating based on polysiloxane modified with different ratios of NAC and crosslinked via UV-assisted thiolene addition	*S. pneumoniae*	Very small numbers of bacteria on the material surface, without signs of biofilm formation on crosslinked siloxane samples without and with NAC
Johnson (2007) [37]	Silastic models with either an intact or a widely opened port, and additionally with either a non-coring needle penetrating the port, or a non-coring needle removed from the port	*S. aureus*	Significantly higher bacterial counts (*p* = 0.0003) in CIs with widely fenestrated ports
Cevizci (2014) [29]	Novel quorum sensing inhibitor, named “yd 47”	*S. pneumoniae*	No biofilm formation by pneumococci in implant materials and at the surrounding soft tissue
Suri (2021) [26]	Gel of polyhexanide and betaine surfactant	*P. aeruginosa, S. aureus, S. epidermidis*	Reduction of surface tension and significant removal of debris and bacteria. Prevention of further growth of biofilm
Brady (2006) [7]	Tea tree oil and terpinen-4-ol	MRSA	Complete prevention of biofilm by 1% tea tree oil. Complete eradication of biofilm by 5% tea tree oil. Sufficient eradication of biofilm by terpinen-4-ol or hydrogen peroxide.
Kao (2017) [22]	Surface modification by using a layer-by-layer polyelectrolyte assembly method	*P. aeruginosa*	Significant decrease in adherence of the bacteria
Höing (2018) [39]	Antibiofilm activity of coating with S53P4 bioactive glass	*Pseudomonas aeruginosa, Staphylococcus aureus, Staphylococcus epidermidis, Streptococcus pyogenes*	Significant reduction of *P. aeruginosa* and *S. aureus* biofilm
Kirchoff (2020) [40]	Antibiofilm activity of coating with S53P4 bioactive glass	*P. aeruginosa, S. aureus*	Significant changes in the morphology of biofilm, visualized by SEM

Abbreviations: CIs = cochlear implants, NPs = nanoparticles, LDP = liquid phase deposition, ALD = atomic layer deposition, NAC = N-acetyl-L-cysteine, UV = ultraviolet radiation, MRSA = methicillin-resistant Staphylococcus aureus.

## Supplementary Materials

The following supporting information can be downloaded at: https://www.mdpi.com/article/10.3390/medicina60121959/s1, Supplementary File S1: Quality assessment of in vitro and animal studies. Supplementary File S2: Quality assessment of case reports. Supplementary File S3: Quality assessment of case series. Supplementary File S4: Quality assessment of non-randomized studies (ROBINS-I tool).
