# Supplementary material for: Development and Prevention of Biofilm on Cochlear Implants: A Systematic Review
Source: Medicina (Kaunas). 2024 Nov 28;60(12):1959. doi: 10.3390/medicina60121959 (PMC11678758; doi:10.3390/medicina60121959)
Supplement: Supplementary file 1 [file medicina-60-01959-s001.zip › medicina-3308744-supplementary.pdf]

	Notan (2016)	Chen (2016)	Cozma (2021)	Johnson (2007)	Cevizci (2014)	Suri (2021)	Brady (2010)	Kao (2017)	Höing (2018)	Kirchoff (2020)	Goldfinger (2014)
**Abstract**	YES	YES	YES	YES	YES	YES	YES	YES	YES	YES	YES
**introduction**	YES	YES	YES	YES	YES	YES	YES	YES	YES	YES	YES
**Background and objectives**	YES	YES	YES	YES	YES	YES	YES	YES	YES	YES	YES
**Methods intervention**	YES	YES	YES	YES	YES	YES	YES	YES	YES	YES	YES
**Outcomes**	YES	YES	YES	YES	YES	YES	YES	YES	YES	YES	YES
**Sample size**	YES	YES	YES	YES	YES	YES	YES	YES	YES	YES	YES
**Randomization: sequence generation**	NO	NO	NO	NO	NO	NO	NO	NO	NO	NO	NO
**Allocation concealment mechanism**	NO	NO	NO	NO	NO	NO	NO	NO	NO	NO	NO
**Implementation**	NO	NO	NO	NO	NO	NO	NO	NO	NO	NO	NO
**Blinding**	NO	NO	NO	NO	NO	NO	NO	NO	NO	NO	NO
**Statistical methods**	NO	YES	NO	YES	NO	NO	NO	YES	YES	NO	NO
**Outcomes and estimation**	YES	YES	YES	YES	YES	YES	YES	YES	YES	YES	YES
**Limitations**	NO	NO	NO	NO	NO	NO	NO	NO	NO	NO	NO
**Funding**	NO	NO	YES	NO	NO	YES	NO	NO	NO	NO	NO
**Protocol**	NO	NO	NO	NO	NO	NO	NO	NO	NO	NO	NO

Supplementary File S1. Quality assessment of *in vitro* and animal studies

	Vaid (2013)	Im (2015)	Celerier (2017)	Cristobal (2004)	Kos (2009)	Skrivan (2016)	Pawlowski (2005)	Fishpool (2012)	Makarem (2008)
Were patient's demographic characteristics clearly described?	YES	YES	YES	YES	YES	YES	YES	YES	YES
Was the patient's history clearly described and presented as a timeline?	YES	YES	YES	YES	YES	YES	YES	YES	YES
Was the current clinical condition of the patient on presentation clearly described?	YES	YES	YES	YES	YES	YES	YES	YES	YES
Were diagnostic tests or assessment methods and the results clearly described?	YES	YES	YES	YES	YES	YES	YES	YES	YES
Was the intervention(s) or treatment procedure(s) clearly described?	YES	YES	YES	YES	YES	YES	YES	YES	YES
Was the post-intervention clinical condition clearly described?	YES	YES	YES	YES	YES	YES	YES	YES	YES
Does the case report provide take away lessons?	YES	YES	YES	YES	YES	YES	YES	YES	YES

Supplementary File S2. Quality assessment of case reports

	Antonelli (2004)	Olsen (2018)	Cunningham (2004)	Suri (2021)
Is the objective of the study stated clearly in the abstract, introduction, or methods section?	YES	YES	YES	YES
Are the characteristics of the participants included in the study described?	YES	YES	YES	YES
Were the cases collected in more than one centre?	NO	NO	NO	NO
Are the eligibility criteria (inclusion and exclusion criteria) explicit and appropriate?	YES	YES	YES	YES
Were patients recruited consecutively?	YES	YES	YES	YES
Did patients enter the study at a similar point in the disease?	YES	YES	YES	YES
Did the authors describe the intervention?	YES	YES	YES	YES
In addition to intervention, did the patients receive any co-interventions?	NO	YES	NO	YES
Was loss to follow-up reported?	NO	NO	NO	NO
Are outcomes (primary, secondary) clearly defined in the introduction or methodology section?	NO	NO	NO	NO
Did the authors use accurate (standard, valid, reliable) objective methods to measure the outcomes?	YES	YES	YES	YES
Were outcomes assessed before and after intervention?	YES	YES	YES	YES
Was the length of follow-up clearly described/reported?	NO	YES	YES	NO
Were the statistical tests used to assess the primary outcomes appropriate?	YES	YES	YES	YES
Does the study provide estimates of the random variability in the data for the primary outcomes (e.g. standard error, standard deviation, confidence intervals)?	NO	YES	NO	NO
Was the analysis of outcomes based on intention to treat?	YES	YES	YES	YES
Are adverse events that may be a consequence of the intervention reported?	NO	NO	NO	NO
Are the conclusions of the study supported by results?	YES	YES	YES	YES
Is there a competing interest statement about the type and source of support received for the study or about the relationship of the author(s) or other contributors with the manufacturer of the technology?	NO	YES	NO	YES

Supplementary File S3. Quality assessment of case series

	Ruellan (2010)
**Bias due to confounding factors**	Low risk
**Bias in selection of participants into the study**	Low risk
**Bias in classification of interventions**	Low risk
**Bias due to deviations from intended interventions**	Low risk
**Bias due to missing outcome data**	Low risk
**Bias in measurement of outcomes**	Low risk
**Bias in selection of reported results**	Low risk
**Overall Bias**	Low risk

Supplementary File S4. Quality assessment of non-randomized studies (ROBINS-I tool)
